# Metropolitan Hospital Sunday Fund

**Published:** 1901-08-03

**Authors:** 


### The Institutional Workshop.

### METROPOLITAN HOSPITAL SUNDAY FUND.

The Lord Mayor (Mr. Alderman Green) presided at a meeting of the Council of the Metropolitan Hospital-Sunday Fund held at the Mansion House on Tuesday, July 30th ult. The annual general report of the Committee of Distribution which was presented stated as follows:—

The Committee of Distribution recommend awards this year to 196 institutions. The amount of collections, including interest from the Court of Chancery, to the present time is £51,000: this sum includes £4,000 sent to the president of our fund by Sir Frederick L. Cook, Bart., with a request that it might be divided equally between the London Hospital, Guy's Hospital, St. Thomas's Hospital, and St. George's Hospital. This course will be adopted and the cash will be paid to these four hospitals, together with the amount of the award recommended by your Committee when confirmed by the Council, and the Committee anticipate a further £1,500 before the accounts are closed on October 31st. The distribution of £44,534 to 142 hospitals, etc., and to 54 dispensaries is now recommended.

This year 200 institutions, or one more than last year, have made applications for grants from the funds at your disposal. Of this number it was found necessary to invite the attendance of deputations from the governing bodies of eighteen. Your Committee are not able to recommend any awards to two dispensaries and one convalescent home; in the latter case the accounts worked out to a minus basis. Deputations from six hospitals and from three dispensaries conferred with your Committee. The remaining nine replied by letter leaving the matter in the hands of your Committee. Explanations modifying the causes of adverse criticism were given in four cases.

The serious dissensions between the Medical Staff and the Board of Management at the National Hospital for Paralysis, Queen Square, have recently been investigated by a committee of inquiry, whose report has been followed by the resignation of the secretary-director, and a decision of the governors of the hospital to admit two of the staff on to the Board of Management. If this restores harmony between the Staff and the Board, the Distribution Committee will at once report to the Council with reference to awards to the National Hospital for the years 1900 and 1901.

Five per cent. of the total sum collected—viz., about £2,500—is set apart to purchase surgical appliances (in obedience to Law XII.), in monthly proportions, during the ensuing year.

In compliance with an order of the Council, and for the special use of its members, tables of statistics have been prepared as usual, showing an analysis of the number of beds in hospitals, the cost of patients both at hospitals and dispensaries, the proportionate expense of management, as well as other valuable information, and copies are sent to every member of the Council.

Mr. George Herring has again most liberally sent a further donation of £10,000. The collections on Hospital Sunday are generally well maintained, and show the confidence the public have in the Metropolitan Hospital-Sunday Fund.

Your Committee recommend that all payments to the fund after this date be carried to the credit of next year's fund.

Before moving the adoption of the report, Sir Sydney Waterlow, the Chairman of the Distribution Committee, mentioned that a letter had been received from the London School Board making a complaint against the Royal Eye Hospital. This letter he suggested should be referred back to the Distribution Committee, it having been received too late for previous consideration, the grant to the Royal Eye Hospital being in the meantime suspended. This was seconded by Dr. Glover, and adopted.

Sir Sydney Waterlow then moved, "That the report of the Committee of Distribution for the year 1901 be approved, and that the several awards recommended be paid as soon as possible, that to the Royal Eye Hospital being referred to the Distribution Committee with the letter from the London School Board, the Committee to act therein as shall seem best." They would sympathise with him, he said, in the pride and pleasure he felt in moving such a resolution for the twenty-ninth time—a statement that was received by the Council with warm and hearty cheers. With reference to the Royal Eye Hospital, he said the School Board complained that when their scholars were sent there wholesale for examination they (the Board) were not treated well by the authorities of the hospital. Well, there had been no time to go into the matter at all, and as it was not wise to legislate on a one-sided statement of a case they had deferred the grant till it could be considered. Turning to the report, he wished the collections increased in the same ratio as the applications for participation did, but he thought that the receipts this year might be considered satisfactory. They had not reached—and he supposed they never would reach—the £100,000 they had set before themselves as the goal of their ambition, but he looked upon it as a great source of satisfaction that a million and a quarter had been collected since the institution of the Fund, and that that sum had been distributed without any complaint of consequence. This year they had 200 institutions applying: they had invited the attendance of the authorities of eighteen of these, and nine had sent letters expressing their willingness to leave the matter in the hands of the Distributing Committee. In other cases they had had long conferences with the deputations sent, and where faults were shown to exist such as ought to be rectified, they had promised to come before them next year with a clean slate. With respect to the National Hospital for Paralysis it would be remembered that last year they had decided not to make an award until the differences that had arisen between the Medical Staff and the Board of Management had been settled; and this year they felt that until they received from the Medical Committee a report that arrangements had been made to satisfy their requirements, and until the new rules were in force, they should make no award. They had enough in reserve to pay last year's award and any other the Council might sanction. He thought the inquiry had done the hospital a great deal of good. There was no hospital in the world with a finer or more brilliant staff, and as soon as the report was received he hoped to trouble the Council with another meeting to get this matter settled. Sir Sydney concluded by expressing the gratitude of the Committee to Mr. George Herring for his continued munificence, and also to Sir Frederick Cook for the gift that had been distributed in accordance with the conditions attached.

Sir Savile Crossley seconded the motion. He was glad to know that all three funds—the Sunday, the Saturday, and the Prince of Wales's—with which he was connected were progressing and were ahead of what they were this time last year. He hoped it would be possible for the secretaries of these three kindred funds to meet and confer as to the forms sent out to hospitals so as to bring them more closely into accord, and so save the time of the hospital secretaries.

Sir Henry Burdett said that with regard to the reference to the National Hospital for Paralysis he should like to submit a question or two. It seemed to him a most desirable thing that in any internal trouble arising in a hospital that committee should be very careful to take no side. They had solely to deal with the efficiency of the management and the provision of funds. The National Hospital stood alone; the demand for its beds was greater than the supply; and he thought it a very grave position to force on the interregnum committee the necessity of having to do without some thirteen or fourteen thousand pounds, and so, possibly, having to close one of their wards. He failed to see what advantage could accrue to anyone by the holding over of the awards. They had had the report of the investigation committee which had entailed the resignation of the secretary-director, and the Committee now acting represented all the parties to the dispute. If they continued to withhold the money it must imply that they considered the Governors or the Committee did not understand their business, and that the final settlement would not be a satisfactory one. He had done all in his power to promote peace in this matter, and he strongly urged that there be no further delay.

The Hon. Sydney Holland cordially supported Sir Henry Burdett. He thought the Governors of the National Hospital had done all they could do. They had had a Committee of Inquiry; they had adopted its decisions; they had appointed a committee to carry out its recommendations. It would be quite impossible for that committee, of which he was a member, to make its report for some time. They had had two meetings; two members of the medical staff had been adopted, and the secretary-director had resigned. All the points in dispute had therefore been settled, and like everyone else they had adjourned till October. Then they would have to put the new rules before counsel; so that it would be Christmas or perhaps January before they could report. It would be a cruel act if the Council were to delay the awards. He would suggest that they should pay over last year's and this year's as well, and then if things did not go right, stop awards in the future. He strongly deprecated crippling the institution by further delay now. Another matter in the report he wished to refer to. He saw that for the first time the Queen's Jubilee Hospital was put down for a grant. He expressed regret that this had been done, since he did not consider that the institution was a proper one to receive aid from that fund, its building and arrangements not being suitable for hospital work: he did not think it was right to call it a hospital in comparison with the others on the list.

The Lord Mayor and Sir Sydney Waterlow both defended the inclusion of the Queen's Jubilee Hospital in the list of grants on the ground that an effort was being made at improvement. A lady had given the institution £1,000 and with this they were beginning to build. The Council therefore thought they should give them some encouragement, since a hospital was certainly wanted in the neighbourhood.

After some further discussion respecting the National Hospital for Paralysis it was agreed to empower the distribution committee to make an award for this year and to request the treasurer to pay it to the hospital forthwith together with the award held over from last year. With this alteration the report was adopted *nem. con.*

The Earl of Stamford moved that the cordial thanks of the Council be given to Sir Sydney Waterlow, the Chairman, and to the other members of the committee of distribution for the care bestowed in the preparation of the awards they had recommended. This was carried with acclamation.

In moving that the thanks of the Council be given to the editors of newspapers who had advocated the cause of the Fund, Mr. Holland expressed the hope that the newspapers would be merciful when anything was reported as wrong in connection with a hospital, and not condemn them without sending to ascertain the other side. As a case in point, he referred to the fact that damage would inevitably result to the Royal Eye Hospital from the fact that it had been mentioned there that day that the School Board had made some complaint with regard to that hospital; though he could bear testimony that if the School Board had behaved to the Royal Eye as it had behaved to the London Hospital, he did not wonder that the authorities of the Royal Eye had resented it; and that he understood was all the charge that was made. So far as the children were concerned, he believed the hospital had done all it possibly could.

The Lord Mayor joined with Mr. Holland in hoping that the Press would not let it be understood that the Council endorsed the letter of the London School Board by their action in deferring the grant; while Sir Sydney Waterlow was anxious it should be known that concerning the work of the hospital no complaint at all was even made.

Dr. Glover also endorsed this view, remarking that if the School Board suddenly, and without warning, sent scores or hundreds of children to be dealt with, the hospital must be held entirely blameless for any disorganisation of its arrangements that might have occurred. He hoped the Council, so far from injuring the hospital, might be able to do it a positive service.

A vote of thanks to the Lord Mayor for his services as President and Treasurer for the year concluded the proceedings.

